# Supplementary material for: Metabolomics during canine pregnancy and lactation
Source: PLoS One. 2023 May 10;18(5):e0284570. doi: 10.1371/journal.pone.0284570 (PMC10171673; doi:10.1371/journal.pone.0284570)
Supplement: Attachment — Submitted filename: PONE-D-22-35093_reviewer.pdf 4.pdf [file pone.0284570.s002.pdf]

PLOS ONE

Metabolomics during canine pregnancy and lactation

--Manuscript Draft--

**Manuscript Number:**	PONE-D-22-35093
**Article Type:**	Research Article
**Full Title:**	Metabolomics during canine pregnancy and lactation
**Short Title:**	Metabolomics during canine pregnancy and lactation
**Corresponding Author:**	Alexander Bartel<br>Freie Universität Berlin Fachbereich Veterinärmedizin: Freie Universitat Berlin Fachbereich Veterinarmedizin<br>Berlin, GERMANY
**Keywords:**	canine metabolism; reproduction; Nursing; physiology during pregnancy and lactation in dogs; nuclear magnetic resonance metabolomics
**Abstract:**	During pregnancy and parturition, female dogs have to cope with various challenges such as providing nutrients for the growth of the fetuses, hormonal changes, whelping, nursing, milk production, and uterine involution. In that regard, it is elementary to know the typical signs of progress and parameters during pregnancy, parturition, and in the puerperium.<br>Metabolomic findings may support a broader understanding of the physiological changes caused by pregnancy that are likely to be key to normal fetal growth and development. Metabolomic research has been used to characterize the metabolic effects of multiple environmental factors, and inter- and intra-individual factors, including feeding, aging, inter-breed differences, drug action, behavior, exercise, genetic factors, and pathologic processes.<br>This study identifies multiple metabolites showing specific changes at the different phases of pregnancy and lactation. Several metabolites such as albumin, glycoprotein acetyls, fatty acids, lipoproteins, glucose, and some amino acids show similar patterns during pregnancy and lactation as shown in humans. The patterns of some other parameters such as branched-chain amino acids, alanine and histidine seem to differ between these species.<br>For most metabolites, it is yet unstudied whether the observed changes arise from modified resorption from the intestines, modified production, or metabolism in the maternal or fetal tissues. Hence, further species-specific research is needed to shed more light on these phenomena. Our findings provide a baseline of normal metabolic changes during canine pregnancy and parturition. Combined with future metabolomics findings, they may help monitor vital functions of pre-, intra-, and post-partum bitches and may allow early detection of illness.
**Order of Authors:**	Sebastian Patrick Arlt
	Claudia Ottka
	Hannes Lohi
	Janna Hinderer
	Julia Lüdeke
	Elisabeth Müller
	Corinna Weber
	Barbara Kohn
	Alexander Bartel
**Additional Information:**	
**Question**	**Response**
**Financial Disclosure**	The author(s) received no specific funding for this work. The costs were covered by the Freie Universitaet Berlin (examoination, sampling) and PetBiomics Ltd (Analyses).

Powered by Editorial Manager® and ProduXion Manager® from Aries Systems Corporation

Enter a financial disclosure statement that describes the sources of funding for the work included in this submission. Review the submission guidelines for detailed requirements. View published research articles from *PLOS ONE* for specific examples.<br><br>This statement is required for submission and **will appear in the published article** if the submission is accepted. Please make sure it is accurate.<br><br>**Unfunded studies**<br>Enter: *The author(s) received no specific funding for this work.*<br><br>**Funded studies**<br>Enter a statement with the following details:<br>• Initials of the authors who received each award<br>• Grant numbers awarded to each author<br>• The full name of each funder<br>• URL of each funder website<br>• Did the sponsors or funders play any role in the study design, data collection and analysis, decision to publish, or preparation of the manuscript?<br>• **NO** - Include this sentence at the end of your statement: *The funders had no role in study design, data collection and analysis, decision to publish, or preparation of the manuscript.*<br>• **YES** - Specify the role(s) played.<br><br>* typeset	
**Competing Interests**<br><br>Use the instructions below to enter a competing interest statement for this submission. On behalf of all authors, disclose any competing interests that could be perceived to bias this work—acknowledging all financial support and any other relevant financial or non-financial competing interests.<br><br>This statement is required for submission and **will appear in the published article** if the submission is accepted. Please make	I have read the journal's policy and the authors of this manuscript have the following competing interests: Claudia Ottka was an employee and Hannes Lohi is a shareholder and the chairman of the board of PetBIOMICS Ltd, who developed and provides the metabolomics test. All other authors declare to have no competing interests.

sure it is accurate and that any funding sources listed in your Funding Information later in the submission form are also declared in your Financial Disclosure statement.<br><br>View published research articles from *PLOS ONE* for specific examples.<br><br>**NO authors have competing interests**<br><br>Enter: *The authors have declared that no competing interests exist.*<br><br>**Authors with competing interests**<br><br>Enter competing interest details beginning with this statement:<br><br>*I have read the journal's policy and the authors of this manuscript have the following competing interests: [insert competing interests here]*<br><br>* typeset	
**Ethics Statement**<br><br>Enter an ethics statement for this submission. This statement is required if the study involved:<br><br>• Human participants<br>• Human specimens or tissue<br>• Vertebrate animals or cephalopods<br>• Vertebrate embryos or tissues<br>• Field research<br><br>Write "N/A" if the submission does not require an ethics statement.<br><br>General guidance is provided below. Consult the submission guidelines for detailed instructions. **Make sure that all information entered here is included in the Methods section of the manuscript.**	Informed written consent and approval<br>from the owners was obtained and sample collection was approved by the committee on animal welfare of the federal state Berlin (LAGeSo Berlin O 0095/18).

**Format for specific study types**<br><br>**Human Subject Research (involving human participants and/or tissue)**<br>• Give the name of the institutional review board or ethics committee that approved the study<br>• Include the approval number and/or a statement indicating approval of this research<br>• Indicate the form of consent obtained (written/oral) or the reason that consent was not obtained (e.g. the data were analyzed anonymously)<br><br>**Animal Research (involving vertebrate animals, embryos or tissues)**<br>• Provide the name of the Institutional Animal Care and Use Committee (IACUC) or other relevant ethics board that reviewed the study protocol, and indicate whether they approved this research or granted a formal waiver of ethical approval<br>• Include an approval number if one was obtained<br>• If the study involved *non-human primates*, add *additional details* about animal welfare and steps taken to ameliorate suffering<br>• If anesthesia, euthanasia, or any kind of animal sacrifice is part of the study, include briefly which substances and/or methods were applied<br><br>**Field Research**<br><br>Include the following details if this study involves the collection of plant, animal, or other materials from a natural setting:<br>• Field permit number<br>• Name of the institution or relevant body that granted permission	
**Data Availability**<br><br>Authors are required to make all data underlying the findings described fully available, without restriction, and from the time of publication. PLOS allows rare exceptions to address legal and ethical concerns. See the PLOS Data Policy and FAQ for detailed information.	Yes - all data are fully available without restriction

A Data Availability Statement describing where the data can be found is required at submission. Your answers to this question constitute the Data Availability Statement and **will be published in the article**, if accepted.<br><br>**Important:** Stating 'data available on request from the author' is not sufficient. If your data are only available upon request, select 'No' for the first question and explain your exceptional situation in the text box.<br><br>Do the authors confirm that all data underlying the findings described in their manuscript are fully available without restriction?	
**Describe where the data may be found in full sentences. If you are copying our sample text, replace any instances of XXX with the appropriate details.**<br><br>• If the data are **held or will be held in a public repository**, include URLs, accession numbers or DOIs. If this information will only be available after acceptance, indicate this by ticking the box below. For example: *All XXX files are available from the XXX database (accession number(s) XXX, XXX.*).<br>• If the data are all contained **within the manuscript and/or Supporting Information files**, enter the following: *All relevant data are within the manuscript and its Supporting Information files.*<br>• If neither of these applies but you are able to provide **details of access elsewhere**, with or without limitations, please do so. For example:<br><br>*Data cannot be shared publicly because of [XXX]. Data are available from the XXX Institutional Data Access / Ethics Committee (contact via XXX) for researchers who meet the criteria for access to confidential data.*<br><br>*The data underlying the results presented in the study are available from (include the name of the third party*	All relevant data are within the manuscript and its Supporting Information files.

and contact information or URL).<br>• This text is appropriate if the data are owned by a third party and authors do not have permission to share the data.<br><br>* typeset	
Additional data availability information:	

Powered by Editorial Manager® and ProduXion Manager® from Aries Systems Corporation

Manuscript
Click here to access/download;Manuscript;Metabolomics_plosone.docx

Metabolomics during canine pregnancy and lactation

Sebastian P. Arlt[1,2*], Claudia Ottka[3], Hannes Lohi[3,4,5], Janna Hinderer[2], Julia Lüdeke[2], Elisabeth Müller[6], Corinna Weber[6], Barbara Kohn[7], Alexander Bartel[8*]

[1]Clinic of Reproductive Medicine, Vetsuisse Faculty, University of Zurich, Zurich, Switzerland

[2]Clinic for Animal Reproduction, Faculty of Veterinary Medicine, Freie Universitaet Berlin, Koenigsweg 65, 14163 Berlin, Germany

[3]PetBiomics Ltd, Helsinki, Finland

[4]Department of Veterinary Biosciences and Department of Medical and Clinical Genetics, University of Helsinki, Helsinki, Finland

[5]Folkhälsan Research Center, Helsinki, Finland

[6]Laboklin GmbH & Co KG, Steubenstrasse 4, 97688 Bad Kissingen, Germany

[7]Clinic for Small Animals, Faculty of Veterinary Medicine, Freie Universitaet Berlin, Oertzenweg 19b, 14163 Berlin, Germany

[8]Institute for Veterinary Epidemiology and Biostatistics, Faculty of Veterinary Medicine, Freie Universitaet Berlin, Koenigsweg 67, 14163 Berlin, Germany

* Corresponding authors

E-mail: sebastian.arlt@uzh.ch (SPA) and alexander.bartel@fu-berlin.de (AB)

Abstract

During pregnancy and parturition, female dogs have to cope with various challenges such as providing nutrients for the growth of the fetuses, hormonal changes, whelping, nursing, milk production, and uterine involution. In that regard, it is elementary to know the typical signs of progress and parameters during pregnancy, parturition, and in the puerperium.

Metabolomic findings may support a broader understanding of the physiological changes caused by pregnancy that are likely to be key to normal fetal growth and development. Metabolomic research has been used to characterize the metabolic effects of multiple environmental factors, and inter- and intra-individual factors, including feeding, aging, inter-breed differences, drug action, behavior, exercise, genetic factors, and pathologic processes.

This study identifies multiple metabolites showing specific changes at the different phases of pregnancy and lactation. Several metabolites such as albumin, glycoprotein acetyls, fatty acids, lipoproteins, glucose, and some amino acids show similar patterns during pregnancy and lactation as shown in humans. The patterns of some other parameters such as branched-chain amino acids, alanine and histidine seem to differ between these species.

For most metabolites, it is yet unstudied whether the observed changes arise from modified resorption from the intestines, modified production, or metabolism in the maternal or fetal tissues. Hence, further species-specific research is needed to shed more light on these phenomena. Our findings provide a baseline of normal metabolic changes during canine pregnancy and parturition. Combined with future metabolomics findings, they may help monitor vital functions of pre-, intra-, and post-partum bitches and may allow early detection of illness.

Introduction

Pregnancy and parturition are challenging for mammals because they are characterized by considerable hormonal and metabolic changes [1,2] to support fetal demands [3,4]. It has been claimed that even small deviations from the norm at different pregnancy stages might have major consequences for the mother and the offspring [5].

In dogs, pregnancy-related changes might be even more intensive than in many other mammalian species because fetal development is rapid as pregnancy duration is, on average, only 63 days, and a bitch might carry a high number of conceptuses. Parturition usually lasts several hours, is laborious, and often exhausting [6]. Other parameters may change during pregnancy and parturition because of hormonal changes, uterine enlargement, pain, anxiety and uterine contractions [7]. In addition, the canine endotheliochorial placentation type leads to a longer and more intensive uterine involution process than other species [8]. Finally, also tasks such as nursing the puppies, including milk production and change of diet, are challenging for the female dog [1].

Understanding the physiology, endocrinology, and metabolic features during pregnancy, parturition, and lactation are required to understand and diagnose normal and abnormal conditions and provide optimal supportive management [1]. In that regard, it is important to realize that some parameters change physiologically during pregnancy, parturition, and lactation [7].

Metabolomics is an omics-based approach that generates comprehensive information about metabolites in blood serum, enabling an extensive view of the individual's current state of systemic metabolism (9, 10). Several metabolomics studies have been conducted in humans to address the physiological and pathophysiological metabolic changes during pregnancy and lactation, but no previous studies have been conducted in dogs. This study aimed to determine the metabolic changes occurring in dogs during pregnancy and lactation and to discuss their similarities or differences to the changes occurring in humans.

Materials and Methods

Animals

In total, 27 privately owned bitches of 21 different breeds were sampled during this study (Table 1). Dogs were enrolled from March 2018 until December 2019. To be included, bitches had to be generally healthy within the past 6 months before mating and not receive medication, except deworming. In addition, dogs had to become pregnant and be presented for sampling at six time-points. Previous gynecological illnesses were inquired about but not present in any of the bitches at the time of samplings. Informed written consent and approval from the owners was obtained and blood sample collection was approved by the committee on animal welfare of the federal state Berlin (LAGeSo Berlin O 0095/18).

Dogs were initially presented for ovulation timing. After they met the inclusion criteria, an initial clinical and gynecological examination was performed, including vaginoscopy and vaginal cytology.

Blood sampling and sample processing

For this study, three stages of canine pregnancy were defined according to Hinderer et al. [9]. A total of six appointments for drawing blood samples were scheduled. The first sample was taken at a visit for ovulation timing in estrus (E). Three samplings during pregnancy were appointed, i. e., between day 11 and 19 (P1) after mating, between day 23 and day 32 (P2), and between day 52 and 60 (P3). The P2 examination included an ultrasonic examination to assert whether the bitch was pregnant. This examination was not scheduled in the middle of the second third to respect the owners' wishes of having a pregnancy diagnosis rather sooner than later while completing the study examination within the same appointment. A fifth sampling was performed 18 to 24 days after whelping. The last clinical examination (A) was performed 18 to 24 days after the weaning of the litter.

All blood samples were collected in the morning or early afternoon.

After parturition, data on the delivery (natural or cesarean section), the number and weight of puppies (alive and stillborn), and survival after three weeks were documented.

Blood samples were collected by venipuncture from either the cephalic or the saphenous vein into plastic tubes (Sarstedt Tube 4.5 mL, Clotting Activator/Serum, 75 × 13 mm, Sarstedt AG & Co KG, Nümbrecht, Germany). Samples were left at room temperature for 30 to 60 min for clotting and then centrifuged at 5000 rpm or 2884 g for 5 min (Hettich Centrifuge EBA 20, Hettich, Tuttlingen, Germany). Serum was transferred into a serum tube (Simport Cryovial sterile with lip seal design, external threads, 2 mL Tubes, Boloeil QC, Canada), which was placed in a refrigerator at – 80°C until shipment. The storage temperature was checked daily, and variations of the measured temperature remained between -80°C and – 83°C. Storage duration at – 80°C of the samples ranged from 11 to 30 months.

Only complete sample sets with sufficient serum and no haemolytic appearance were submitted for NMR.

For shipment preparation, the tubes were thawed for one to three hours, and then an amount of 0.3ml was transferred into a second serum tube (Simport Cryovial sterile with lip seal design external threads, 2 mL Tubes, Boloeil QC, Canada), both aliquots were again immediately frozen at – 80°C. Overnight shipment to PetBiomics, Finland, was performed in a Styrofoam box with plenty of dry ice.

NMR metabolomics analyses

A validated and highly automated canine-specific NMR metabolomics technology was utilized for metabolomics analyses of the collected serum samples [10]. The method quantitates 123 measurands from various molecular groups, including a comprehensive lipoprotein analysis, fatty acids, triglycerides, cholesterols, amino acids, glycolysis- and fluid balance-related metabolites, and a novel inflammatory marker glycoprotein acetyls (GlycA) [10]. A similar method has been largely utilized in human metabolomics studies [11–13], including a study on the metabolic effects of human pregnancy [2]. Technical details of the method are provided elsewhere [11–13] .

Statistical analysis

All statistical analyses were performed using R version 4.1.3 (R Foundation for Statistical Computing, Vienna). First, the data were checked for missing observations. The data did not include any metabolite values missing at random. Values below the detection limit were treated as zero values in the following statistical analyses.

For metabolite selection, a Friedman-Test (a non-parametric alternative to a repeated-measures ANOVA) was used to determine which metabolite concentrations showed significant changes between the different time points and thus are affected by the course of the pregnancy.The p-value for the effect of the time points on each metabolite was calculated, using a random effect to account for the measurement of each animal at every of the 6 time points. All p-values were corrected for multiple comparisons using the Benjamini-Hochberg method, and the p-value cutoff for adjusted p-values was set at $p<0.05$.

KMeans Clustering was done using the z-scaled values of each metabolite. The optimal number of clusters was determined using the “gap” statistic (R package “cluster” version 2.1.3, [14]). Cluster were afterward modified based on the biochemical relationships between the metabolites (super/subcategories, absolute/relative values of the same metabolite). To visualize the effect of the time points on the metabolites, “locally estimated scatterplot smoothing” (LOESS) was used. The reference intervals of the analysis [9] were added to the plots to provide reference on the typical concentrations of the metabolites and to highlight the magnitude of the change.

Results

The 27 dogs enrolled in the study (Table 1) belonged to 21 different breeds and had a median body weight of 22.8 kg (Min: 9.0, Q1: 15.1, Q3: 32.6, Max: 56.4kg). The median age was 4.35 years (Min 1.98, Q1: 2.9, Q3: 5.77, Max 8.1 years).

Table 1. Attributes of female dogs included in a study on metabolimics during canine pregnancy and lactation

Dog No	Breed	Bodyweight (kg)	Parity*	Age	Number of puppies born	Thereof stillborn puppies
**1**	Dogue de Bordeaux	51,3	4	7.0	2	0
**2**	Miniature Bull Terrier	16,8	1	2.9	6	3
**3**	Rhodesian Ridgeback	38,0	2	4.9	12	1
**4**	Saarloos Wolfhound	28,8	2	6.2	5	0
**5**	Collie	24,0	2	6.3	5	0
**6**	Borzoi	36,4	1	8.1	8	0
**7**	Labrador Retriever	22,4	1	2.6	9	0
**8**	Staffordshire Bull Terrier	14,2	1	2.6	1	0
**9**	French Bulldog	11,8	2	2.6	3	1
**10**	Schnauzer	18,2	2	7.8	6	0
**11**	Olde English Bulldogge	32,2	1	2.0	12	1
**12**	Miniature Bull Terrier	19,0	2	4.7	7	0
**13**	Wirehaired Dachshund	9,0	2	4.9	7	0
**14**	Boxer	27,8	2	6.8	4	0
**15**	Hovawart	41,6	2	6.7	9	1
**16**	Irish Wolfhound	56,4	1	2.9	13	1
**17**	Golden Retriever	22,8	1	4.4	5	0
**18**	Entlebucher Mountain Dog	23,6	1	2.6	7	0
**19**	Golden Retriever	33,0	1	4.6	6	0
**20**	Miniature Bull Terrier	16,0	2	5.4	6	1
**21**	Eurasier	20,8	2	3.8	5	0
**22**	Wirehaired Dachshund	9,2	1	3.4	6	0
**23**	Kromfohrlaender	10,4	1	2.9	7	1
**24**	English Cocker Spaniel	11,2	2	2.9	8	0
**25**	Labrador Retriever	28,2	2	5.3	6	0
**26**	English Cocker Spaniel	11,2	2	2.9	6	0
**27**	Dobermann	38,8	1	3.4	13	0

* including the current pregnancy

Fig 1 shows the metabolic parameters which were high in mid-pregnancy (P2). Metabolites which showed different patterns, i. e. low in mid-pregnancy, high in late pregnancy, high in lactation, and low in lactation are presented in Fig 2 (cluster 2, 3,4,5).

Fig 1. Metabolic parameters high in mid-pregnancy (Cluster 1) Change in metabolic parameters over the course of canine pregnancy (E = estrus, P1-3 = early/mid/late pregnancy, L = lactation, A = after weaning). Points show the individual measurements. The solid line shows the change in average metabolite levels (LOESS). Dashed lines show the upper and lower limits of the metabolite reference intervals [9]. The p-values were calculated using a Friedman-Test with Benjamini-Hochberg correction. The p-value cutoff for adjusted p-values was $p < 0.05$.

Fig 2. Metabolic parameters low in mid-pregnancy, high in late pregnancy, high in lactation, and low in lactation (Cluster 2,3,4,5) Change in metabolic parameters over the course of canine pregnancy (E = estrus, P1-3 = early/mid/late pregnancy, L = lactation, A = after weaning). Points show the individual measurements. The solid line shows the change in average metabolite levels (LOESS). Dashed lines show the upper and lower limits of the metabolite reference intervals [9]. The p-values were calculated using a Friedman-Test with Benjamini-Hochberg correction. The p-value cutoff for adjusted p-values was $p < 0.05$.

Discussion

Metabolism during pregnancy and lactation is a dynamic and precisely programmed process [5], as this stage of life is defined by rapid fetal or neonatal growth and development [2,15]. Metabolite adaptations, such as increased hepatic glucose output, decreased peripheral insulin sensitivity, unchanged hepatic insulin sensitivity and lower peripheral insulin levels, allow the dam to maintain euglycemia during pregnancy [16] and to support fetal growth despite the increasing nutritional demands [17]. Nevertheless, nutrition should be adapted to the respective pregnancy phase to ensure the health of the dam and the puppies [18]. It has been shown that protein deposition in maternal and fetal tissues increases throughout pregnancy, most occurring during the third trimester [15]. Furthermore, leptin increased proportionally with increased food intake during pregnancy, although an apparent body weight gain was observed only at day 60 [19]. The concentrations of metabolites in the blood serum of the bitch affect the corresponding concentrations and the composition of amniotic and allantoic fluid [17]. These fluids are essential

during pregnancy, providing metabolites, protection, and the environment for normal fetal development [20].

Five distinct clusters were observed in the analysis of the patterns of the assessed parameters. The identified clusters were: parameters high (Cluster 1/Figure 1)/low (Cluster 2/Figure 2) in mid-pregnancy, parameters high at late pregnancy (Cluster 3/Figure 2), and parameters high (Cluster 4/Figure 2)/low (Cluster 5/Figure 2) in lactation. The clustering was based on similar patterns in metabolite concentrations with elevations and decreases in metabolite concentration occurring during similar time points within each cluster.

Cluster 1: parameters high in mid-pregnancy

Total fatty acids tended to be higher during pregnancy than at other time points, albeit the differences were not significant. However, an increase in the molar concentration of individual fatty acids was also observed, which may explain changes in the total fatty acids. In addition, elevated total fatty acids have also been observed in human pregnancies because a high concentration of fatty acids is necessary to meet the demands of fetuses [3]. Fatty acids play an important role in the development of the fetus and may also be involved in modulatory effects on the mother's immune system [21].

The concentration of **free cholesterol (significant)** was highest in mid-pregnancy. The same pattern was mirrored in **total cholesterol** and **esterified cholesterol** (both not significant). These findings are not following another study assessing 12 dogs assigned to four different diet groups in which plasma total cholesterol concentrations were depressed in early gestation and then increased in the later stages independently of the diet [22]. These differences refer mostly to a sample taken on day 42. At this time point, we did not sample the dogs. It is well known that cholesterol also rises during human pregnancies [23].

Lipoproteins are divided into four major classes: chylomicrons, very low-density lipoproteins (VLDL), low-density lipoproteins (LDL), and high-density lipoproteins (HDL), with HDL being the most abundant lipoprotein in dogs [24]. Chylomicrons and VLDL are the main carriers of serum triglycerides, while HDL and LDL contain mainly cholesterol [25].

The lipoprotein parameters show different patterns in the course of pregnancy and lactation in the dog. While the concentration of HDL particles (HDL-P) and VLDL particles (VLDL-P) show

no changes, many others, such as **HDL size**, **total lipids in very large HDL (XL-HDL total lipids)**, the **concentration of LDL particles (LDL particles), LDL cholesterol, total lipids in LDL (LDL total lipids)** peak in mid-pregnancy and decline after that. Other lipoprotein parameters peak in late pregnancy (see cluster 3). It is well known that dogs have a unique lipid metabolism and produce substantial, cholesterol-rich HDL particles in hypercholesterolemia. Other than in dogs, in humans, these cholesterols are usually transformed into the more atherogenic LDL particles, which is believed to increase the risk of atherosclerosis in humans [25].

The percentage of polyunsaturated fatty acids (**PUFA %**) (significant) and **PUFA** (not significant) were high in mid-pregnancy. The same phenomenon has been shown in humans [3,21]. In humans, polyunsaturated fatty acids (PUFA) accumulate in maternal fat depots in early pregnancy and become available for placental transfer during late pregnancy when the fetal growth rate is maximal and fetal requirements for PUFAs are greatly enhanced [26].

Omega-3 unsaturated fatty acids and **omega-3%** were high in mid-pregnancy (both significant), as well as **omega-6** (not significant) and **omega-6%** (significant). Omega-3 and Omega-6 PUFA are needed to ensure the physiologic development of nervous tissue, retinal tissue, kidney, liver, and skin functions [22].

Docosahexaenoic acid (DHA) belongs to Omega-3 fatty acids and was significantly elevated in mid-pregnancy, as well as **DHA%**. It plays a significant role during pregnancy and lactation because it contributes to brain development, accounting for over 10% of brain fatty acids [27]. It has been shown that DHA increases during gestation in humans, but its proportion of total lipids decreases [28]. The authors suggest that this indicates a preferential transfer of DHA across the placenta to the fetus [28].

Arachidonic acid (AA), an Omega-6 fatty acid, and AA% were significantly elevated in mid-pregnancy. At the same time, **linoleic acid (LA**), also an Omega-3 fatty acid and precursor for DHA and Arachidonic acid, was only slightly elevated in dogs. It has been shown that concentrations of LA and AA also increase in pregnant women [28].

Total saturated fatty acids (**SFA**), including **palmitic acid (PalA)** and **stearic acid (SteA),** were slightly elevated during pregnancy in dogs. In humans, the increase of SFA and PalA is markedly higher until the third trimester [21].

Glycoprotein acetyls (GlycA) is a novel spectroscopic marker of systemic inflammation combining the *N*-acetyl methyl group proton signals of several acute-phase proteins. It peaks significantly in mid-pregnancy in dogs. In humans, the intra-individual biological variation of this marker is low, and the main acute-phase protein contributors to its signal are α1-acid glycoprotein, haptoglobin, α1-antitrypsin, and α1-antichymotrypsin [29]. It has been shown that GlycA is higher in pregnant women than in non-pregnant women [3]. The placentation process might explain the rise of this parameter and peak during mid-pregnancy. It is well known that acute phase proteins such as fibrinogen and c-reactive protein increase from day 30 to 50 [30]. A rise of acute phase proteins during pregnancy has also been shown in 12 pregnant bitches [31].

These findings follow the research on eight pregnant Beagle dogs, which has shown that Alpha-1-acid glycoprotein, which contributes to the GlycA signal, rises during dog pregnancy and peaks at around 45 days of gestation" [32]. In humans, Alpha-1-antitrypsin, another contributor to the GlycA signal, increases in pregnancy [33].

The only amino acid which shows a marked peak during mid-pregnancy is **phenylalanine**. In humans, it has been demonstrated that phenylalanine, alanine, and histidine were increased in pregnant women [3]. In dogs, alanine and histidine seem not to increase during pregnancy. Phenylalanine is required for protein synthesis and is intracellularly converted to tyrosine, which, in turn, is either used for protein synthesis, oxidized, or converted to the important neurotransmitters epinephrine, norepinephrine, and dopamine [34]. In rats, it has been shown that excessive feeding of phenylalanine to the dams impairs proliferation and hypertrophy in the fetal brain [35], but the dietary requirement for phenylalanine in healthy human pregnancies or canine pregnancies has not been determined yet. Dietary phenylalanine requirements seem not to vary between adult and non-pregnant dog breeds [36].

Cluster 2: parameters low in mid-pregnancy

Oleic acid is a monounsaturated omega-9 fatty acid. Compared to other unsaturated fatty acids (see Cluster 1), its concentration slightly declines in mid-pregnancy. In humans, Oleic acid increases during pregnancy [21].

Free cholesterol and **phospholipids** in large VLDL (L-VLDL) and **total lipids in extensive VLDL (XL-VLDL total lipids)** decline significantly during mid-pregnancy and increase towards the end of pregnancy. An increase in all parameters during pregnancy has been observed in humans [37].

Cluster 3: parameters high in late pregnancy

Through various mechanisms, pregnancy causes insulin resistance, suppressing the intracellular transport of glucose and increasing blood glucose concentrations in dogs [4,38]. Glucose concentrations significantly increase during late pregnancy and remain high until the time after weaning. Despite the pronounced insulin resistance in pregnant compared with non-pregnant diestrous bitches [30], gestational diabetes mellitus in dogs is rare [39]. The same mechanisms of insulin resistance have been described in humans [40], albeit several groups have found lower glucose concentrations in pregnant women compared with non-pregnant ones [3]. The mechanism causing this decline is not well understood. Potential contributing factors include dilutional effects, an increased utilization by the fetoplacental unit or increased maternal uptake secondary to increased β-cell function, and/or inadequate hepatic production [40].

Total triglycerides, LDL Triglycerides, and VLDL triglycerides significantly peak in late pregnancy. This is in accordance with findings in normal human pregnancies. Shen et al. [41] found that the levels of lipids, including TG, TC, and LDL cholesterol, increased gradually during gestation and peaked before delivery; meanwhile, HDL cholesterol amounts increased from the 1st to 2nd trimester with a slight decrease in the 3rd trimester. Furthermore, in human pregnancies, an abnormal increase in triglycerides during pregnancy is associated with gestational diabetes mellitus [41]. The possible disease correlations of abnormally high triglycerides during canine pregnancy require further study. Similar to gestational diabetes mellitus, hypertensive disorders are not common complications of canine pregnancy. Interestingly, unlike humans, dogs' HDL triglycerides are not affected by pregnancy or lactation.

Cluster 4: parameters high in lactation

It has been shown that lactate and pyruvate are more elevated in pregnant women than in non-pregnant women [3]. The **lactate, pyruvate,** and **acetate** concentrations significantly rise towards late pregnancy and lactation, presumably reflecting the higher demands of energy metabolism, even if their medians remain within the reference intervals at all time points. Whether lactate can be used as an indicator for sepsis or other pregnancy-related disorders in dogs as it is used in humans [42] requires further study since all bitches included in our study had undisturbed pregnancies.

The total concentration of **branched-chain amino acids (BCAA),** which includes leucine, isoleucine, and valine, shows a marked increase during lactation. Interestingly, isoleucine and valine show the same pattern, whereas leucine shows low concentrations during lactation (see cluster 5). The same is true for the amino acid **glycine**. Glutamine, glycine, valine, and tyrosine were lower in pregnant women compared with non-pregnant women; no differences were found for isoleucine and leucine between pregnant and non-pregnant women [3].

Creatinine shows a significant decrease in mid-pregnancy, which is in accordance with findings in pregnant women. It has been stated that a physiologic increase in the glomerular filtration rate during pregnancy results in a decrease in the concentration of serum creatinine [43]. In addition, it has been shown that creatinine was lower in pregnant women compared to non-pregnant women [3].

Cluster 5: parameters low in lactation

The observed significant decline of **albumin** during late pregnancy and lactation has also been well documented in humans during and after normal pregnancies [3,44]. It is believed that this decline is caused by physiologic hemodilution but may lead to physiological edema and even may associate with conditions such as eclampsia [44]. In addition, it has been shown that albumin concentrations increase in the allantoic fluid in late canine pregnancy [20].

Docosapentaenoic acid (DPA) is also significantly elevated during pregnancy but shows a significant decline during lactation, which is also visible using the parameter **DPA%**.

The concentration of the amino acid **histidine** remains stable at all time points, except for a significant decrease during lactation. Histidine likely plays a role in milk production, as it has been shown for cows, who have an increased energy-corrected milk yield when supplemented with histidine [45].

The amino acid **leucine** showed a significant increase in its concentration during early pregnancy and a decline during mid and late pregnancy and lactation. This contrasts with other branched-chain amino acids (see cluster 4). This decline is likely connected with milk production since leucine plays a vital role in milk production, as shown in cows [46].

Limitations of the study

The metabolome is affected by several internal factors such as gene and protein activity and external factors, including diet and environmental factors [47]. In our study, privately owned dogs were used, which were housed and fed under different conditions. Furthermore, 21 breeds and dogs aged between 2 and 8 years were included. To what extent these heterogeneous conditions may have affected the results remains open. However, the included dogs may better reflect the real-life heterogeneity seen in pregnant dogs than laboratory animals. Experienced breeders kept most dogs so that appropriate housing and feeding under consideration of the reproductive status of the female dog is likely.

A further limitation is that dogs did not fast before sampling because this would not have been acceptable for pregnant and lactating dogs. The timing of collection of the blood samples also showed a mild variation of a few hours; therefore, the impact of diurnal influence cannot be excluded. However, as they were usually sampled in the morning, diurnal variance would be expected to be small. Another potential limitation is the small number of dogs originating from one geographical area.

Some comparisons of our findings with findings from human pregnancies need to be interpreted with caution. Pregnancy of dogs is around 63 days, significantly shorter, and the offspring usually is much larger. Puppies are born physiologically immature and dependent on their mother´s care and milk supply [48]. In the study, the dogs delivered between 1 and 12 puppies.

In addition, research on pregnant dogs has led to several contradictory findings, which makes interpretation of actual metabolic and hematologic findings difficult [1]. For example, it was

described more than 40 years ago that some bitches develop progressive, normochromic, and normocytic anemia beginning in mid-pregnancy [6]. While some authors consider this normal, other groups suggest that haematocrits should remain in the normal reference range and that bitches with anaemia should be examined for other concurrent diseases [49].

The samples for this study were taken throughout the year. However, previous research using the same method has shown that only minimal seasonal variation exists in the vast majority of metabolites [50]. Therefore, the season was not taken into account during the data analysis.

In our current study, samples were stored at −80°C for up to 30 months before analysis. In a previous study validating the NMR platform, all studied metabolites were stable at −80°C for at least 12 months [10]. However, the impact of more extended storage on canine samples is lacking.

In many species, it can be challenging to determine whether changes detected during pregnancy are related to the presence of a conceptus or the increased progesterone concentrations [31].

Conclusions

Several metabolites such as albumin, GlycA, fatty acids, lipoproteins, glucose, and some amino acids show similar patterns during canine pregnancy and lactation as shown in humans. The patterns of some other parameters, such as branched-chain amino acids, alanine and histidine, seem to differ.

To date, it is unclear for most metabolites if observed changes arise from modified resorption from the intestines, modified production or metabolism in the maternal tissues, or their migration through the placenta. Hence, further species-specific research is needed to shed more light on these questions.

Understanding the molecular changes during pregnancy and the relation to discrete biological events such as embryo development and placentation may support understanding of these processes, learn more about causes of infertility or diseases, and aid in identifying treatments for these conditions [50]. It is essential to understand physiological changes during pregnancy and lactation so that these changes are not misinterpreted as indicators of disease or malnutrition. Furthermore, knowledge of the physiological changes is important for designing

studies on metabolites to adjust inclusion criteria, i. e. if or not to include also pregnant or lactating bitches, and for interpreting the results.

Further research should provide molecular reference ranges in normal pregnancies in relation to studies of adverse pregnancy outcomes [3]. In that regard, metabolomics findings may help monitor vital functions of pre-, intra-, and post-partum bitches and early detection of illness [1].

Acknowledgments

The authors wish to thank the participating breeders for their cooperation and interest in the study.

References

1. Arlt SP. The bitch around parturition. Theriogenology. 2020; 150:452–7. Epub 2020/03/04. doi: 10.1016/j.theriogenology.2020.02.046 PMID: 32164993.
2. Cardinali L, Troisi A, Verstegen JP, Menchetti L, Elad Ngonput A, Boiti C, et al. Serum concentration dynamic of energy homeostasis hormones, leptin, insulin, thyroid hormones, and cortisol throughout canine pregnancy and lactation. Theriogenology. 2017; 97:154–8. Epub 2017/04/28. doi: 10.1016/j.theriogenology.2017.04.040 PMID: 28583599.
3. Wang Q, Würtz P, Auro K, Mäkinen V-P, Kangas AJ, Soininen P, et al. Metabolic profiling of pregnancy: cross-sectional and longitudinal evidence. BMC Med. 2016; 14:205. Epub 2016/12/13. doi: 10.1186/s12916-016-0733-0 PMID: 27955712.
4. Johnson CA. Glucose homeostasis during canine pregnancy: Insulin resistance, ketosis, and hypoglycemia. Theriogenology. 2008; 70:1418–23. Epub 2008/09/27. doi: 10.1016/j.theriogenology.2008.09.008 PMID: 18823654.
5. Liang L, Rasmussen M-LH, Piening B, Shen X, Chen S, Röst H, et al. Metabolic Dynamics and Prediction of Gestational Age and Time to Delivery in Pregnant Women. Cell. 2020; 181:1680-1692.e15. doi: 10.1016/j.cell.2020.05.002 PMID: 32589958.
6. Concannon PW, Powers ME, Holder W, Hansel W. Pregnancy and parturition in the bitch. Biol Reprod. 1977; 16:517–26.
7. Lúcio CF, Silva LCG, Rodrigues JA, Veiga GAL, Vannucchi CI. Peripartum Haemodynamic Status of Bitches with Normal Birth or Dystocia // Peripartum haemodynamic status of bitches with normal birth or dystocia. Reprod Dom Anim. 2009; 44 // 44 Suppl 2:133–6. doi: 10.1111/j.1439-0531.2009.01427.x PMID: 19754552.
8. Schulze LS-C, Heuwieser W, Arlt SP. Body temperature of bitches in the first week after parturition measured by ingestible loggers. Reprod Domest Anim. 2018; 53 Suppl 3:63–9. doi: 10.1111/rda.13330 PMID: 30474328.
9. Hinderer J, Lüdeke J, Riege L, Haimerl P, Bartel A, Kohn B, et al. Progesterone Concentrations during Canine Pregnancy. Animals (Basel). 2021; 11. Epub 2021/11/24. doi: 10.3390/ani11123369 PMID: 34944146.
10. Ottka C, Vapalahti K, Puurunen J, Vahtera L, Lohi H. A novel canine nuclear magnetic resonance spectroscopy-based metabolomics platform: Validation and sample handling.

Vet Clin Pathol. 2021; 50:410–26. Epub 2021/08/24. doi: 10.1111/vcp.12954 PMID: 34431130.

11. Soininen P, Kangas AJ, Würtz P, Tukiainen T, Tynkkynen T, Laatikainen R, et al. High-throughput serum NMR metabonomics for cost-effective holistic studies on systemic metabolism. Analyst. 2009; 134:1781–5. Epub 2009/07/30. doi: 10.1039/b910205a PMID: 19684899.

12. Soininen P, Kangas AJ, Würtz P, Suna T, Ala-Korpela M. Quantitative serum nuclear magnetic resonance metabolomics in cardiovascular epidemiology and genetics. Circ Cardiovasc Genet. 2015; 8:192–206. doi: 10.1161/CIRCGENETICS.114.000216 PMID: 25691689.

13. Würtz P, Kangas AJ, Soininen P, Lawlor DA, Davey Smith G, Ala-Korpela M. Quantitative Serum Nuclear Magnetic Resonance Metabolomics in Large-Scale Epidemiology: A Primer on -Omic Technologies. Am J Epidemiol. 2017; 186:1084–96. doi: 10.1093/aje/kwx016 PMID: 29106475.

14. Maechler M, Rousseeuw P, Struyf A, Hubert M, Hornik K. cluster: Cluster Analysis Basics and Extensions. ; 2022.

15. Elango R, Ball RO. Protein and Amino Acid Requirements during Pregnancy. Adv Nutr. 2016; 7:839S-44S. Epub 2016/07/15. doi: 10.3945/an.115.011817 PMID: 27422521.

16. Connolly CC, Papa T, Smith MS, Lacy DB, Williams PE, Moore MC. Hepatic and muscle insulin action during late pregnancy in the dog. Am J Physiol Regul Integr Comp Physiol. 2007; 292:R447-52. Epub 2006/09/14. doi: 10.1152/ajpregu.00385.2006 PMID: 16973936.

17. Balogh O, Bruckmaier R, Keller S, Reichler IM. Effect of maternal metabolism on fetal supply: Glucose, non-esterified fatty acids and beta-hydroxybutyrate concentrations in canine maternal serum and fetal fluids at term pregnancy. Anim Reprod Sci. 2018; 193:209–16. Epub 2018/04/20. doi: 10.1016/j.anireprosci.2018.04.072 PMID: 29716779.

18. Greco DS. Nutritional supplements for pregnant and lactating bitches. Theriogenology. 2008; 70:393–6. Epub 2008/06/12. doi: 10.1016/j.theriogenology.2008.04.013 PMID: 18554708.

19. Troisi A, Cardinali L, Menchetti L, Speranza R, Verstegen JP, Polisca A. Serum concentrations of leptin in pregnant and non-pregnant bitches. Reprod Domest Anim. 2020; 55:454–9. Epub 2020/02/20. doi: 10.1111/rda.13637 PMID: 31957075.

20. Tal S, Sutton GA, Arlt SP, Bar-Gal GK. Analysis of biochemical parameters in canine fetal fluids during the second half of pregnancy. Theriogenology. 2022; 189:31–41. Epub 2022/05/20. doi: 10.1016/j.theriogenology.2022.05.009 PMID: 35716430.

21. Aparicio E, Martín-Grau C, Hernández-Martinez C, Voltas N, Canals J, Arija V, et al. Changes in fatty acid levels (saturated, monounsaturated and polyunsaturated) during pregnancy. BMC Pregnancy Childbirth. 2021; 21:778. Epub 2021/11/17. doi: 10.1186/s12884-021-04251-0 PMID: 34789176.

22. Wright-Rodgers AS, Waldron MK, Bigley KE, Lees GE, Bauer JE. Dietary fatty acids alter plasma lipids and lipoprotein distributions in dogs during gestation, lactation, and the perinatal period. J Nutr. 2005; 135:2230–5. doi: 10.1093/jn/135.9.2230 PMID: 16140903.

23. Bartels Ä, O'Donoghue K. Cholesterol in pregnancy: a review of knowns and unknowns. Obstet Med. 2011; 4:147–51. Epub 2011/07/28. doi: 10.1258/om.2011.110003 PMID: 27579113.

24. Maldonado EN, Romero JR, Ochoa B, Aveldaño MI. Lipid and fatty acid composition of canine lipoproteins. Comp Biochem Physiol B Biochem Mol Biol. 2001; 128:719–29. doi: 10.1016/s1096-4959(00)00366-3 PMID: 11290454.

25. Xenoulis PG, Steiner JM. Lipid metabolism and hyperlipidemia in dogs. Vet J. 2010; 183:12–21. Epub 2009/01/23. doi: 10.1016/j.tvjl.2008.10.011 PMID: 19167915.

26. Herrera E, Amusquivar E, López-Soldado I, Ortega H. Maternal lipid metabolism and placental lipid transfer. Horm Res. 2006; 65 Suppl 3:59–64. Epub 2006/04/10. doi: 10.1159/000091507 PMID: 16612115.

27. Innis SM. Impact of maternal diet on human milk composition and neurological development of infants. Am J Clin Nutr. 2014; 99:734S-41S. Epub 2014/02/05. doi: 10.3945/ajcn.113.072595 PMID: 24500153.

28. Wilson NA, Mantzioris E, Middleton PT, Muhlhausler BS. Gestational age and maternal status of DHA and other polyunsaturated fatty acids in pregnancy: A systematic review. Prostaglandins Leukot Essent Fatty Acids. 2019; 144:16–31. Epub 2019/04/18. doi: 10.1016/j.plefa.2019.04.006 PMID: 31088623.

29. Connelly MA, Otvos JD, Shalaurova I, Playford MP, Mehta NN. GlycA, a novel biomarker of systemic inflammation and cardiovascular disease risk. J Transl Med. 2017; 15:219. Epub 2017/10/27. doi: 10.1186/s12967-017-1321-6 PMID: 29078787.

30. Concannon PW, McCann JP, Temple M. Biology and endocrinology of ovulation, pregnancy and parturition in the dog. J Reprod Fertil Suppl. 1989; 39:3–25.

31. Holst BS, Gustavsson MH, Johannisson A, Hillström A, Strage E, Olsson U, et al. Inflammatory changes during canine pregnancy. Theriogenology. 2019; 125:285–92. Epub 2018/11/15. doi: 10.1016/j.theriogenology.2018.11.016 PMID: 30497027.

32. Kuribayashi T, Shimizu M, Shimada T, Honjyo T, Yamamoto Y, Kuba K, et al. Alpha 1-acid glycoprotein (AAG) levels in healthy and pregnant beagle dogs. Exp Anim. 2003; 52:377–81. doi: 10.1538/expanim.52.377 PMID: 14625401.

33. Lisowska-Myjak B, Sygitowicz G, Wolf B, Pachecka J. Serum alpha-1-antitrypsin concentration during normal and diabetic pregnancy. Eur J Obstet Gynecol Reprod Biol. 2001; 99:53–6. doi: 10.1016/s0301-2115(01)00356-6 PMID: 11604186.

34. Ennis MA, Rasmussen BF, Lim K, Ball RO, Pencharz PB, Courtney-Martin G, et al. Dietary phenylalanine requirements during early and late gestation in healthy pregnant women. Am J Clin Nutr. 2020; 111:351–9. doi: 10.1093/ajcn/nqz288 PMID: 31758682.

35. Manabe S, Inui K, Uenishi K. Effect of excess phenylalanine diet during pregnancy on fetal brain growth in rats. Tokushima J Exp Med. 1993; 40:125–35.

36. Mansilla WD, Gorman A, Fortener L, Shoveller AK, Mansilla WD, Gorman A, et al. Dietary phenylalanine requirements are similar in small, medium, and large breed adult dogs using the direct amino acid oxidation technique. // Dietary phenylalanine requirements are similar in small, medium, and large breed adult dogs using the direct amino acid oxidation technique. J Anim Sci. 2018; 96:3112–20. doi: 10.1093/jas/sky208 PMID: 29846616.

37. Knopp RH, Warth MR, Charles D, Childs M, Li JR, Mabuchi H, et al. Lipoprotein metabolism in pregnancy, fat transport to the fetus, and the effects of diabetes. Biol Neonate. 1986; 50:297–317. doi: 10.1159/000242614 PMID: 3542067.

38. Wada T, Hori S, Sugiyama M, Fujisawa E, Nakano T, Tsuneki H, et al. Progesterone inhibits glucose uptake by affecting diverse steps of insulin signaling in 3T3-L1 adipocytes. Am J Physiol Endocrinol Metab. 2010; 298:E881-8. Epub 2010/01/13. doi: 10.1152/ajpendo.00649.2009 PMID: 20071559.

39. Armenise A, Pastorelli G, Palmisano A, Sontas HB, Romagnoli S, Armenise A, et al. Gestational Diabetes Mellitus with Diabetic Ketoacidosis in a Yorkshire Terrier Bitch // Gestational diabetes mellitus with diabetic ketoacidosis in a Yorkshire terrier bitch. J Am

Anim Hosp Assoc. 2011; 47:285–9. Epub 2011/06/14. doi: 10.5326/Jaaha-Ms-5668 PMID: 21673334.

40. Lain KY, Catalano PM. Metabolic changes in pregnancy. Clin Obstet Gynecol. 2007; 50:938–48. doi: 10.1097/GRF.0b013e31815a5494 PMID: 17982337.

41. Shen H, Liu X, Chen Y, He B, Cheng W. Associations of lipid levels during gestation with hypertensive disorders of pregnancy and gestational diabetes mellitus: a prospective longitudinal cohort study. BMJ Open. 2016; 6:e013509. Epub 2016/12/23. doi: 10.1136/bmjopen-2016-013509 PMID: 28011814.

42. Albright CM, Ali TN, Lopes V, Rouse DJ, Anderson BL. Lactic acid measurement to identify risk of morbidity from sepsis in pregnancy. Am J Perinatol. 2015; 32:481–6. Epub 2014/12/08. doi: 10.1055/s-0034-1395477 PMID: 25486284.

43. Maynard SE, Thadhani R. Pregnancy and the kidney. J Am Soc Nephrol. 2009; 20:14–22. Epub 2008/12/31. doi: 10.1681/ASN.2008050493 PMID: 19118150.

44. Sufrin S, Nessa A, Islam MT, Das RK, Rahman MH. Study on Serum Albumin in Third Trimester of Pregnancy. Mymensingh Med J. 2015; 24:464–6.

45. Räisänen SE, Lage CFA, Oh J, Melgar A, Nedelkov K, Chen X, et al. Histidine dose-response effects on lactational performance and plasma amino acid concentrations in lactating dairy cows: 1. Metabolizable protein-adequate diet. J Dairy Sci. 2021; 104:9902–16. Epub 2021/06/05. doi: 10.3168/jds.2021-20188 PMID: 34099283.

46. Bequette BJ, Metcalf JA, Wray-Cahen D, Backwell FR, Sutton JD, Lomax MA, et al. Leucine and protein metabolism in the lactating dairy cow mammary gland: responses to supplemental dietary crude protein intake. J Dairy Res. 1996; 63:209–22. doi: 10.1017/S002202990003171X PMID: 8861345.

47. Puurunen J, Ottka C, Salonen M, Niskanen JE, Lohi H. Age, breed, sex and diet influence serum metabolite profiles of 2000 pet dogs. R Soc Open Sci. 2022; 9:211642. Epub 2022/02/16. doi: 10.1098/rsos.211642 PMID: 35223061.

48. Tønnessen R, Borge KS, Nødtvedt A, Indrebø A. Canine perinatal mortality: a cohort study of 224 breeds. Theriogenology. 2012; 77:1788–801. Epub 2012/02/25. doi: 10.1016/j.theriogenology.2011.12.023 PMID: 22365700.

49. Cramer KGM de, Joubert KE, Nöthling JO. Hematocrit changes in healthy periparturient bitches that underwent elective cesarean section. Theriogenology. 2016; 86:1333–40. Epub 2016/05/04. doi: 10.1016/j.theriogenology.2016.04.075 PMID: 27242181.

50. Burnum-Johnson KE, Baker ES, Metz TO. Characterizing the lipid and metabolite changes associated with placental function and pregnancy complications using ion mobility spectrometry-mass spectrometry and mass spectrometry imaging. Placenta. 2017; 60 Suppl 1:S67-S72. Epub 2017/03/29. doi: 10.1016/j.placenta.2017.03.016 PMID: 28392013.

Supporting information

S1 File. Metabolomics Raw Data.

Figure 1 Click here to access/download;Figure;2022-08-10_figure1.eps

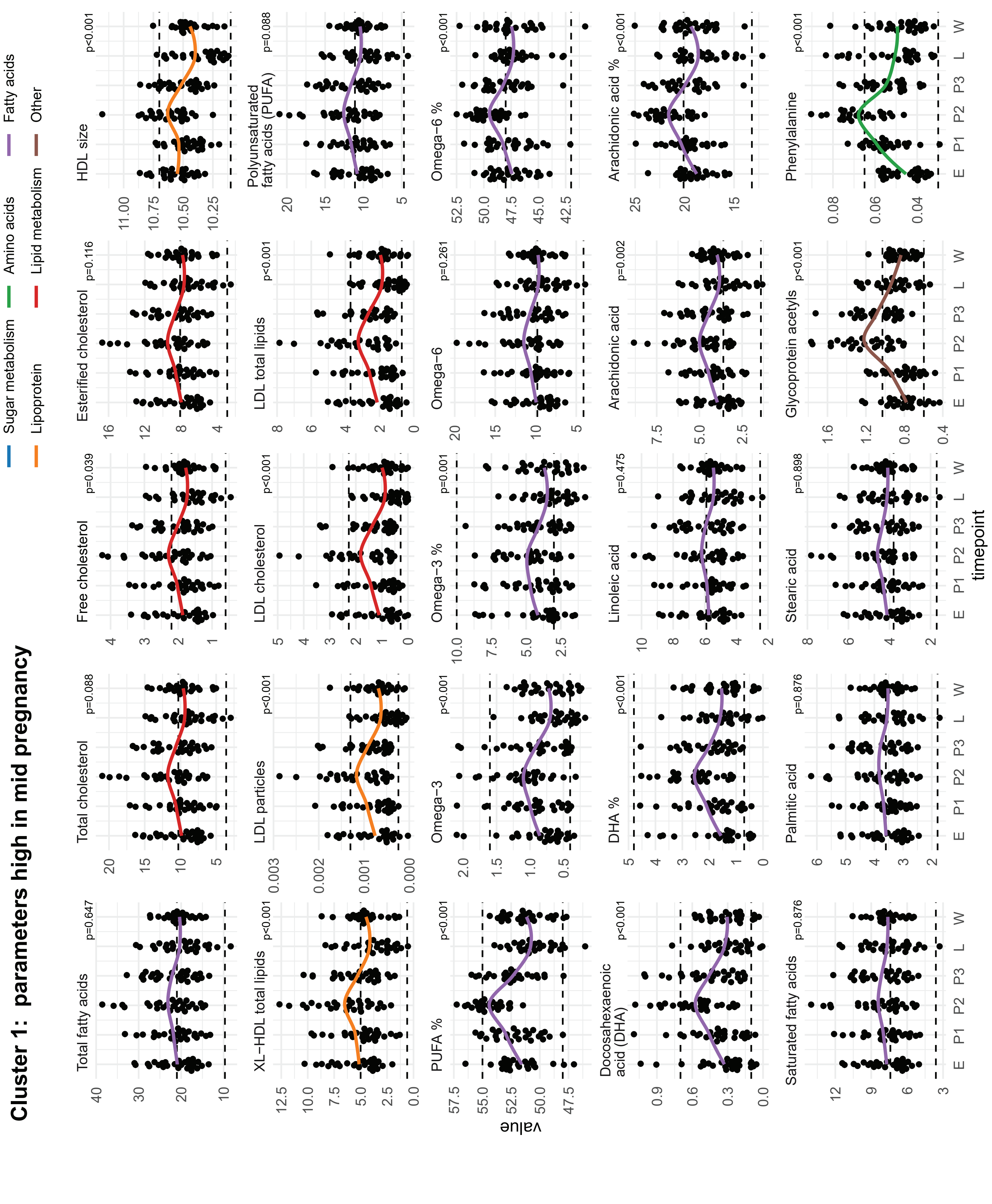

Figure 2

Click here to access/download;Figure;2022-08-10_figure2.eps

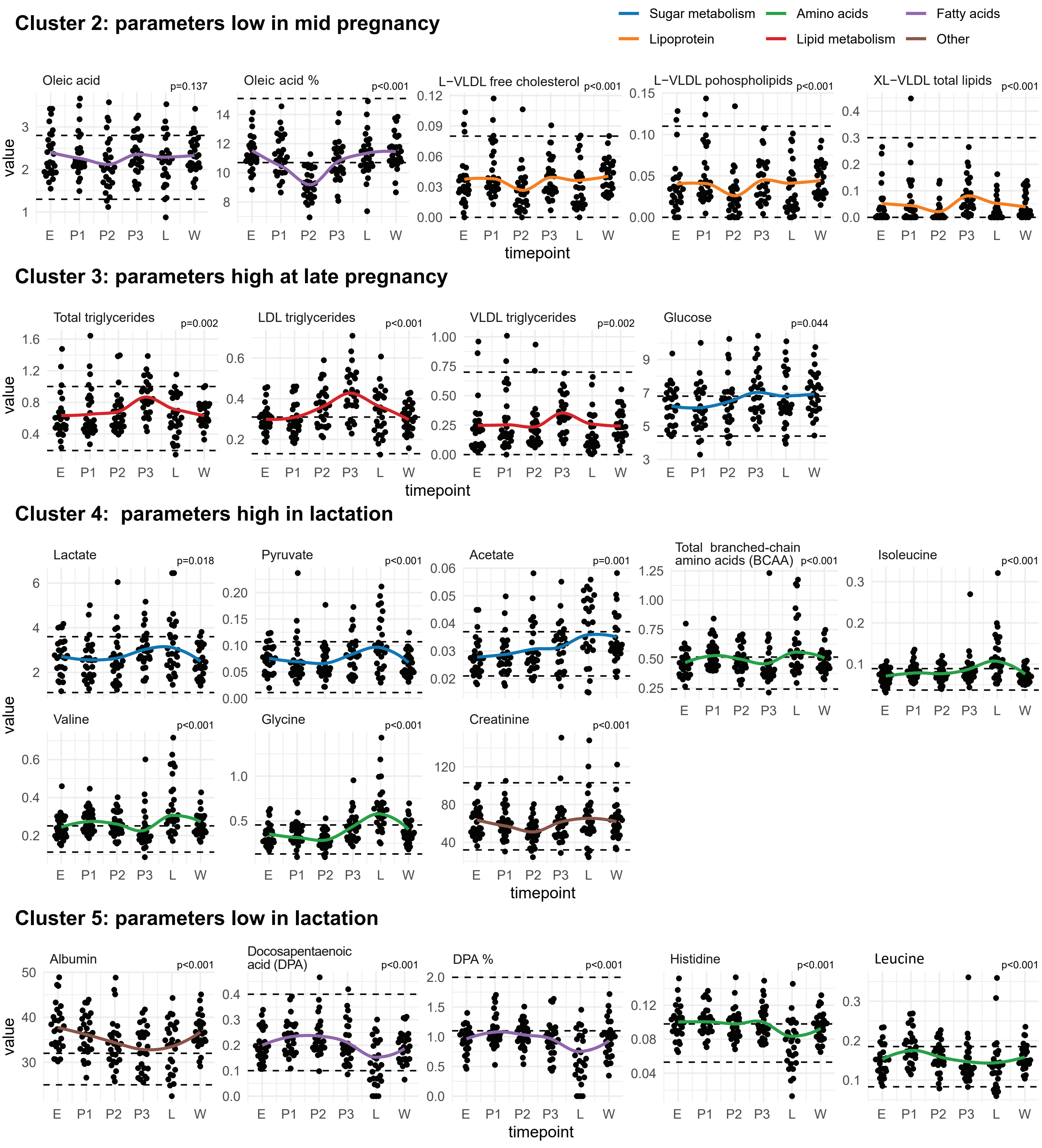

Click here to access/download
Supporting Information
canine_pregnancy_metabolimics_raw_data.csv
